# Personal

**Published:** 1908-03

**Authors:** 


### PERSONAL.

Dr. Gustav A. Hitzel, of Buffalo, was recently elected president of the German-American Alliance, vice Dr. William Gaertner, whose term has expired and who declined reëlection. Dr. Hitzel is a native of Buffalo, a graduate of the University here and has served a term as interne at the Buffalo General Hospital. He has been an active member of the German-American Alliance from the first and it is predicted that the society will make substantial progress under his administration.

---

Dr. John A. Weidman, of Dunkirk, has been reappointed health officer of that village.

---

Dr. Richard C. Cabot, of Boston, recently visited Buffalo and addressed the Men's club of Trinity church on the subject of psychotherapy, the new cult of the Boston Emanuel church. Dr. Charles G. Stockton entertained guests at dinner at the Saturn club Monday evening, February 17, in honor of Dr. Cabot.

---

Major Louis L. Seaman, M.D., of New York, who served as a medical officer in the Spanish war, delivered a lecture on Tuesday evening, February 11, 1908, before the Buffalo Historical Society, on Some of the Curses of Colonisation.

---

Champe S. Andrews, Esq., for so long a time counsel of the Medical Society of the County of New York, has resigned that office and A. C. Vandiver has been appointed to succeed Mr. Andrews. Mr. Vandiver has retired from the staff of District Attorney Jerome and has associated himself with former Judge Charles S. Whitman in the general practice of law at 32 Nassau Street, New York.

---

Dr. Benjamin A. Gipple has been elected a member of the board of trustees of the village of Alden, Erie County, N. Y.

---

Dr. Robert Tuttle Morris was elected president of the Medical Association of Greater New York at its last annual meeting. At a stated meeting of the association held February 17, 1908, Dr. Morris delivered an address the subject of which was, Metaplasia of the Appendix Vermiformis and a New Diagnostic Point.

---

Dr William S. Ely, of Rochester, read a paper before the Rochester Academy of Medicine Wednesday, February 19, 1908, entitled, Human Asymmetry.

---

Dr. Ross G. Loop was elected president of the Elmira Academy of Medicine at its regular meeting held February 5, 1908.

---

Dr. F. Park Lewis, of Buffalo, will address the first International Congress for the Welfare of the Child, to be held under the auspices of the National Mother's Congress, at Washington, March 10-17, 1908. The subject of Dr. Lewis's address is The Prevention of Blindness.

